# Lithotripsy

**Published:** 1846-03

**Authors:** 


*Lithotripsy.*—Calculus measuring one inch and an eighth in diameter, and composed of phosphate of lime—destroyed in eighteen sittings by Heurteloup's instrument, and causing neither pain nor the loss of one drop of blood. By Paul F. Eve, M. D., Professor of Surgery in the Medical College of Georgia.—In my previous communications on the subject of stone in the bladder, published in the American Journal of the Medical Sciences, and in this Journal, the operation recommended was the bilateral. All the patients upon whom I have thus operated were under eight years of age. It gives me great pleasure to state that the first adult laboring under calculous affection, who has applied for relief, has been cured by the bloodless and painless operation of lithotripsy.

Mr. James M. Layton, aged 34, of Early county, in this State, was kindly directed to me by Dr. Wm. J. Johnson, of Fort Gaines. He had been laboring under symptoms of stone for three years, and ten months ago was sounded by Dr. J., and its existence clearly ascertained. Mr. L. arrived here on the 14th of last November, and the next day, after introducing a large sound and finding an ample urethra and good condition of the bladder, lithotripsy was proposed instead of lithotomy, for which he had come to consult me. On the 15th, the calculus was seized with Heurteloup's crusher, and found to measure thirteen and a half lines, or one inch and an eighth in diameter: this was verified by Prof. Means. Having made the necessary preparations, the operation was performed on the 17th, before the Class of the Medical College. The patient was seated on the edge of a table, the instrument introduced, and the stone secured. By gradual forcible pressure with the hand, it suddenly yielded with a crushing report audible to many in the room. It gave the sensation of a pretty resisting shell containing more friable materials. To the gratification of all present, the patient immediately voided some of the debris, one or two fragments being the size of buck-shot. The bladder contained about eight ounces of urine. The operation itself did not occupy five minutes. After it, Mr. Layton amused himself with the students in the College museum; he then walked home to the Surgical Infirmary, took a warm hip-bath, and passed a comfortable night.

On the 18th, at 6, A. M., found his pulse 66 in the recumbent position. He had experienced no pain, and had passed other portions of the stone. Prescribed diluent drinks, moderate diet, and the warm hip-bath twice a day. 6, P. M.—Had eaten some chicken from misunderstanding the directions; pulse at 84.

19th. Not so well this morning; feels some soreness along the urethra; has some fullness about the head, and pulse still 84. To take a dose of calcined magnesia at once, and to use the bath. 3, P. M.—Medicine has operated well and to the entire relief of the patient. Came before the Class again; injected 7 ℥ of mucilage into the bladder; introduced the instrument and crushed two fragments.

From this time to December the 5th, the instrument was employed for a few minutes nearly every day. On the 27th of November, the fragments which had been preserved, (for many had been lost as the bowels had been evacuated at different places, and the smaller particles never were collected,) weighed 47 grains. On the 28th, those passed after one sitting weighed 15 grains. December 2d.—What had been collected for the three past days amounted to 65 grains; and for the two last sittings we weighed 55 grains. Total weight of large fragments, exclusive of some known to have been lost, and of the finer particles, 182 grains, or over ʒiii.

On the 4th, I could detect no stone. On the 5th, I made a careful and minute exploration of the bladder, by all the ordinary processes, without finding a particle remaining—the patient insisting he was entirely free of all symptoms. The next day he was examined by three physicians before the class, and pronounced free of calculous affection. He left the same day for his residence, distant about three hundred miles.—*Southern Medical and Surgical Journal.*

